# Profiling the cell diversity and tissue structure of aqueous humor circulatory system in human eyes using spatial single-cell RNA sequencing

**DOI:** 10.1016/j.gendis.2024.101304

**Published:** 2024-04-12

**Authors:** Hao Yuan, Zhaona Song, Xiao Sun, Chunping Song, Lidong Guo, Qiang Zhang, Yonglun Luo, Chengfu Yuan, Jianlu Gao, Xiaodong Jia

**Affiliations:** a Joint Laboratory for Translational Medicine Research, Liaocheng People's Hospital, Liaocheng, Shandong 252000, China; b College of Life Sciences, University of Chinese Academy of Sciences, Beijing 100049, China; c Department of Ophthalmology, Liaocheng People's Hospital, Liaocheng, Shandong 252000, China; d Lars Bolund Institute of Regenerative Medicine, Qingdao-Europe Advanced Institute for Life Sciences, BGI-Qingdao, BGI-Shenzhen, Qingdao, Shandong 266555, China; e Department of Biomedicine, Aarhus University, Aarhus 8000, Denmark; f Liaocheng People's Hospital, Shandong University, Liaocheng, Shandong 252000, China; g College of Basic Medical Science, China Three Gorges University, Yichang, Hubei 443002, China

Elevated intraocular pressure (IOP) is recognized as a significant contributor to the development of various ocular diseases, especially glaucoma. The delicate balance between the generation and drainage of aqueous humor (AH) in the anterior chamber angle regulates the real IOP level. Despite extensive research, our understanding of the cellular components, tissue structure, and functional heterogeneity within the AH circulatory system (AHCS) remains incomplete, hindering the progression of effective and accessible treatment strategies for IOP intervention. Therefore, the state-of-the-art spatiotemporal single-cell omics stands poised to furnish an invaluable spatial cellular map of the AHCS, thereby laying the foundation for subsequent in-depth investigations. Meanwhile, this innovative approach promises to offer novel insights into the pathogenesis and management of IOP regulation. Here, we utilized nanoscale resolution-spatial enhanced resolution omics-sequencing (Stereo-seq^1^) to obtain *in situ* gene expression profiles of AHCS in human eyes (Fig. S1A). We generated two optimal cutting temperature compound-embedded chips of the trabecular meshwork (TM) and surrounding tissue from four samples (Table S1), which were later cut into layers of 10-μm-thickness cryosections for Stereo-seq and hematoxylin-eosin staining. Considering that transcript capture was performed at a subcellular level using a DNA nanoball sequencing technique, we integrated a semi-automated spatial omics methodology with cell segmentation using the GEM3D-toolkit (https://github.com/BGI-Qingdao/GEM3D_toolkit) to acquire a single-cell resolved transcript of AHCS in spatial scenarios. This method allowed us to partition two Stereo-seq chips into 60,638 putative single cells by assigning transcripts to each defined cell area at single-cell resolution (detailed in supplementary methods and materials and Fig. S1B). After the following quality control, we finally detected 978.5 DNA nanoball spots per cell area on average, with the per-cell detection of 885.4 unique molecular identifiers and 445.3 genes. Chip1 and Chip2 were composed of 32,081 and 28,557 cells, respectively, covering 24,775 genes (Fig. S1C and Table S2).

Subsequently, we independently annotated the cell type for each chip based on its gene expression patterns. Through the utilization of canonical marker genes (Table S3), a total of 15 shared cell clusters across both chips were identified. These encompassed smooth muscle, iris pigment epithelium, ciliary muscle, ciliary body epithelium, corneal epithelium, TM cells, fibroblasts, Schwann cells, erythrocytes, conjunctiva, vessel endothelium, mast cells, melanocytes, stromal cells, and small amount cells of unknown type (Fig. 1A; Fig. S2A, B). Among them, TM was a reticulate structure located in the anterior chamber angle and played a crucial role in controlling the outflow of AH. We verified the enrichment signature scores of TM marker genes and located the area of TM cells in the whole transcriptomics map, consistent with previous anatomical views at the iridocorneal angle^2^ (Fig. 1B, C). Moreover, we discovered a population of low-quality stromal cells, characterized by fewer unique molecular identifiers and lower levels of gene capture, which corresponded to the collagen-covered sparse corneal and scleral regions in the analyzed samples (Fig. S2C). We filtered this cell cluster and the cells of unknown type in downstream analyses.

**Figure 1:**
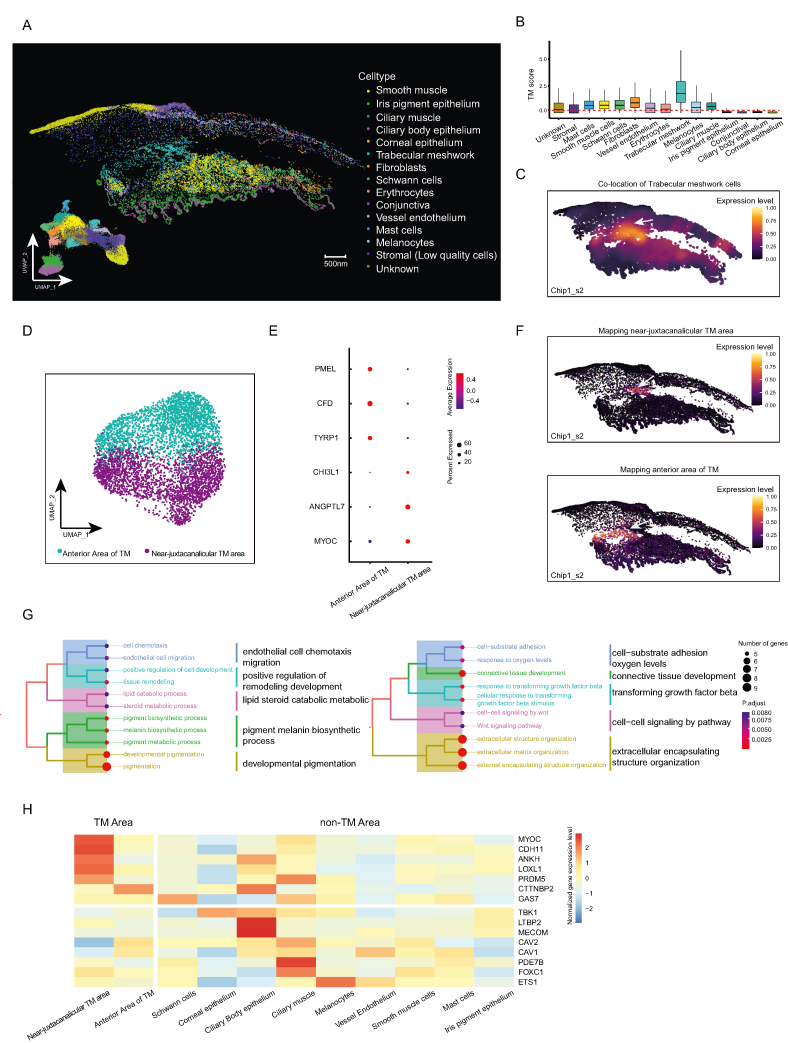
Spatial transcriptome of aqueous humor circulatory system (AHCS) in human eyes. **(A)** Major cell types of AHCS identified by spatial transcriptome and the uniform manifold approximation and projection (UMAP) plot of these cell types (left bottom). chip1_s2 is a representative example. **(B)** The boxplots showing the enrichment signature of trabecular meshwork (TM) marker genes (composed of *MYOC*, *APOD*, and *MGP*) across the whole AHCS cell type. The *Y* axis shows the signature scores for TM cells. Colors denote individual cell clusters. **(C)** Spatial expression of selected TM marker genes (*MYOC*, *APOD*, and *MGP*) in representative chip1_s2 to identify the TM area's tissue location. The spectrum of color represents the mean expression levels of the marker genes. To improve the contrast ratio, a loess fit offered by SPATA2 was used to smooth the expression values. **(D)** UMAP plot of individual TM cells. Blue-green points show the anterior area of TM cells. Purple points show the near-juxtacanalicular area of TM cells. **(E)** The dot plot showing the 6 signature gene expressions across the TM cellular clusters. The size of the dots represents the proportion of cells expressing the particular markers, and the spectrum of color indicates the mean expression levels of the markers (log1p transformed). **(F)** Spatial expression of marker genes of TM subpopulations in representative chip1_s2. The spectrum of color represents the mean expression levels of the marker genes. *CFD* and *TYRP1* were used to map the anterior area of TM in the whole spatial transcriptome; *CHI3L1*, *ANGPTL7*, and *MYOC* were used to map the near-juxtacanalicular TM area. **(G)** The tree plot showing the hierarchical clustering of gene ontology enriched terms. The size of the dots represents the ratio of genes expressing the particular pathways, and the spectrum of color indicates the significance of the current pathway. **(H)** The heatmap showing cell type-specific expression patterns of several representative genes implicated in glaucoma (as detailed in Fig. S3F).

To further elucidate the heterogeneity of the TM area, the harmony algorithm was employed to integrate cell subcluster analysis of TM cells from two distinct chips. We identified the anterior TM area and the near-juxtacanalicular TM area as distinct functional portions (Fig. 1D). The former showed high expression levels of fibrosis-related and melanin production markers such as *CFD* and *TYRP1*, while the latter exhibited canonical expression of AH outflow-related functional markers including *CHI3L1*, *ANGPTL7*, and *MYOC* (Fig. 1E; Figs. S3A–C and Table S3). Our feature genes' mapping analysis revealed significant spatial heterogeneity between two groups of cells, with the near-juxtacanalicular TM subcluster having a smaller cell count and connection to Schwann cells (Fig. 1F). The anterior TM subcluster, located at the forefront of the near-juxtacanalicular subcluster, may serve as a transitory zone for AH outflow. Further immunohistochemical staining using specific antibodies against *CFD* and *MYOC* validated these findings in adjacent TM regions (Fig. S3D). The results indicated that *CFD* expression was higher in the anterior TM region relative to the near-juxtacanalicular region, while *MYOC* showed the reverse pattern. Quantitative analysis suggested similar proportions among different chips for almost all cell types (Fig. S3E). On the other hand, specific TM gene expression patterns consistently corresponded to their functional specificity in different regions (Fig. 1G and Table S4). Our findings of the gene enrichment analysis suggested that the anterior TM region was involved in crucial biological functions such as cell-substrate adhesion, connective tissue formation, sensitivity to transforming growth factor-beta and oxygen levels, and cell–cell WNT signaling. The near-juxtacanalicular TM region played a significant role in heightened pigment melanin biosynthesis and lipid steroid catabolic metabolism, which were essential for the development and homeostasis of the TM subcluster. Additionally, the near-juxtacanalicular area of TM cells was reported to secrete various factors including chemokines, degradative enzymes, and protein components of the extracellular matrix, supporting prolonged extracellular matrix remodeling and modified trabecular cell activity.^3^ Our results revealed that this subcluster had similar functional enrichment, emphasizing the relevance of chemotactic migration and tissue remodeling pathways in controlling IOP homeostasis.

To ascertain the expression pattern of glaucoma-associated genes and meet the future requirements for precise cellular targeted therapy, we employed spatial profiling to examine the expression patterns of certain specific genes across various cell types. Specifically, we considered well-known monogenic causes (Mendelian genes) such as *ANGPT1* and *TBK1*, as well as genes implicated in risk factors from genome-wide association study (GWAS), such as *TMCO1* and *TXNRD2*. Also, we screened an additional 93 genes from the online Mendelian inheritance in man (OMIM) dataset.^4^ A total of 111 genes were selected, each expressed in at least 10% of cells in every cell type, serving as a filtering criterion. The respective expression profiles of these genes are depicted in Figure 1H, with the comprehensive list available in Figure S3F. The genes subjected to testing are detailed in Table S5. As Figure 1H shows, several susceptibility genes associated with high IOP, such as *MYOC*, *CDH11*, and *LOXL1*, were found to be strongly expressed in the near-juxtacanalicular TM cells, demonstrating their cell-specific expression. There were also genes such as *ANKH*, *PRDM5*, and *CTTNBP2*, which were expressed in TM cells as well as in non-TM cells. It is noteworthy that genes such as *GAS7* (expressed in Schwann cells), *EST1* (expressed in melanocytes), and *CAV1/CAV2* (expressed in both endothelial types, ciliary muscle, and vessel endothelium) demonstrated elevated expression levels in the non-TM region compared with the TM region. *TBK1* and *FOXC1*, which were highly heritable features and significant risk factors for the progression of congenital glaucoma and normal-tension glaucoma, showed varied regional expression patterns. *TBK1* was primarily localized to the ciliary body epithelium, whereas *FOXC1* was preferentially localized to the ciliary muscle, followed by the near-juxtacanalicular TM cells and smooth muscle cells. Thus, these results suggested that while abnormalities in the TM region were evident contributors to increased IOP, genes that regulate IOP may not act just within the TM. Cell-targeted therapy necessitated consideration of the specific cells in which the edited gene was expressed. In addition to exploring genes associated with glaucoma, we sought to scrutinize the specific gene expression patterns of extracellular matrix components within the anterior chamber angle, specifically including collagens, fibronectin, laminins, elastin, and fibrillin microfibrils (Fig. S3G). This exploration was prompted by existing literature suggesting their implication in the pathogenesis, progression, and treatment of glaucoma.^5^ We observed the selective expression of many genes encoding collagens and fibronectin, such as *COL6A3*, *COL1A1*, and *FN1*, in the near-juxtacanalicular TM cells. However, the expression of *COL1A2* was noted in both the TM cells of the near-juxtacanalicular region and the ciliary body epithelium. Regarding the encoding genes of laminins and elastin and fibrillin microfibrils, most were uniquely expressed in the epithelium or ciliary muscle. Only *LAMC3* was predominantly expressed in the anterior TM cells. Finally, given the potential of our dataset to offer robust support for future research into the tissue structure and functional dynamics of AHCS, unveiling mechanisms underlying ocular diseases and potential therapeutic approaches, we provided a website link for interactive exploration of our data to expand our research: https://www.bgiocean.com/humaneye/AHCS/.

In conclusion, by employing Stereo-seq, our study provided a valuable spatial single-cell map of AHCS. The approach of data processing utilized in this study could serve as a prospective case for subsequent spatial-omics research endeavors. Moreover, the crucial TM organization within the AH outflow pathway was identified into two distinct functional and spatially heterogeneous subpopulations, namely the anterior TM area cells and the near-juxtacanalicular TM area cells. The gene expression pattern mapping of glaucoma-related and potential therapeutic pathways provided compelling evidence for future targeted cell therapies. An interactive exploration website was also established for accessing the data resources.

## Ethics declaration

This study was consistent with the Declaration of Helsinki and was approved by the ethics committee of Liaocheng People's Hospital, Shandong, China (Approval No. 2021198). Written informed consent has been obtained from the patient and her family.

## Author contributions

X.D.J., J.L.G., and H.Y. conceptualized and designed the study. Z.N.S., X.S. and Q.Z. acquired the data. H.Y., C.P.S., Z.N.S., and L.D.G. analyzed and interpreted the data. H.Y. and X.D.J. were associated with writing, review, and/or revision of the paper. C.F.Y. and Y.L.L. provided technical or material support.

## Conflict of interests

The authors declared no competing financial interests.

## Funding

This work was partially supported by grants from the 10.13039/501100007129Natural Science Foundation of Shandong Province, China (No. ZR2019QH009).

## Data availability

The data and scripts supporting the findings of this study have been deposited into CNSA (CNGB Sequence Archive) of CNGBdb (https://db.cngb.org/cnsa/): Stereo-seq data, CNP0004755.
