# Supplementary material for: Profiling the cell diversity and tissue structure of aqueous humor circulatory system in human eyes using spatial single-cell RNA sequencing
Source: Genes Dis. 2024 Apr 12;12(1):101304. doi: 10.1016/j.gendis.2024.101304 (PMC11472072; doi:10.1016/j.gendis.2024.101304)
Supplement: Multimedia component 1 [file mmc1.pdf]

Profiling the cell diversity and tissue structure of aqueous humor circulatory system in human eyes using spatial single-cell RNA sequencing

Supplementary Information

Supplementary note

The brief introduction of the aqueous humor circulatory system in human eyes

The aqueous humor (AH) circulatory system (AHCS) is a critical cellular niche associated with the establishment and maintenance of intraocular pressure (IOP), which is composed by ciliary body endothelium, the trabecular meshwork (TM), Schlemm canal (SC) and ciliary muscle in turn[1]. As shown in Fig.S1A, AH is secreted by the ciliary body endothelium, circulates within the anterior chamber, most of which flow out of the eye through the TM cells[2], and finally enter the venous system through a network of collector channels. The elevation of IOP is widely acknowledged to be primarily attributed to the obstructed secretion of AH, as opposed to an excessive production[3]. Hence, the dysfunction of TM cells is also generally considered as the primary factor for the regulation and treatment of elevated IOP.

Patients and samples

A patient affected by choroidal melanoma of the right eye with a tumor located in the posterior portion of the eye and a normal anterior eye segment elected to undergo enucleation to remove the affected eyeball. After surgery was complete, we excised the contralateral normal corneoscleral rim for immediate processing. Patient details are provided in Table S1. This study was consistent with the Declaration of Helsinki and was approved by the ethics committee of Liaocheng People's Hospital, Shandong province. The patient and her family provided informed consent.

Tissue preparation for spatial transcriptomic analyses

The surgically dissected tissues were utilized to prepare a tissue block with an edge length of less than 1 cm. Subsequently, this block underwent a cold PBS rinse, was immersed in a pre-chilled tissue storage solution (Miltenyi Biotec, Germany), and was embedded using optimal cutting temperature (OCT) compound (Sakura, USA) in a −30°C microtome (Thermo Fisher, USA) within 30 minutes of completing the surgical procedures. For nanoscale resolution-spatial enhanced resolution omics-sequencing (Stereo-Seq) library preparation, H&E staining, and immunohistochemistry (IHC) analyses, a total of four serial 10 μm cryosections were cut from the OCT-embedded sample. Histopathological analyses of H&E stained samples were conducted using a Motic microscope scanner (Motic, China).

Quality control of RNA obtained from OCT-embedded samples

100–200 μm thick sections were cut from each OCT-embedded sample for total RNA extraction using the RNeasy Mini Kit (Qiagen, USA) according to the manufacturer's protocol. RNA integrity number (RIN) was determined using a 2100 Bioanalyzer (Agilent, USA). Only samples with RIN ≥ 7 were qualified for the transcriptomic study. All samples had an RIN of 7–10 in our study.

Stereo-seq library preparation and sequencing

The spatial transcriptomic RNA library was constructed using Stereo-seq capture chips (BGI-Shenzhen, China) with a size of 1 cm^2. The capture spots were 220 nm in diameter, with a center-to-center distance of 500 nm. Each Stereo-seq capture probe contained a 25 bp

coordinate identity barcode (CID), a 10 bp molecular identity barcode (MID), and a 22 bp poly-T tail for *in situ* mRNA hybridization. The cryosections obtained from OCT-embedded tissue, were promptly positioned on the chip, followed by a 3-minute incubation at 37 °C and subsequent fixation in pre-cooled methanol at −20 °C for 40 minutes. After fixation, the tissue sections underwent staining with Qubit ssDNA dye (Thermo Fisher, USA) to assess tissue integrity before fluorescent imaging. Permeabilization of the tissue sections was achieved using 0.1% pepsin (Sigma, USA) in 0.01 mol/L HCl buffer, with an incubation period of 14 minutes at 37 °C, followed by washing with 0.1× SSC. RNA released from the permeabilized tissue was subjected to reverse transcription for 1 hour at 42 °C. Subsequently, the tissue sections underwent digestion with a tissue removal buffer at 42 °C for 30 minutes. The cDNA-containing chip was then treated with cDNA-release enzyme overnight at 55 °C. The released cDNA was further amplified using a cDNA HIFI PCR mix (MGI). Approximately 20 ng of cDNA was fragmented into 400–600 bp, amplified for 13 cycles, and purified to generate a DNA nanoball library. This library was finally sequenced using the single-end 50 + 100 bp strategy on an MGI DNBSEQ sequencer (MGI, China).

Spatial transcriptomic data processing

Spatially resolved single-cell RNA-seq data generated by Stereo-seq were pre-processed for subsequent analyses. Briefly, read 1 of the sequenced library contains the CIDs, MIDs and poly-T sequences, while corresponding read 2 provides cDNA sequences captured at the same location. The X-Y coordinates (500-nm resolution) of cDNA were interpreted by the CID sequences with 1-bp mismatch tolerance. cDNA sequences were aligned to the reference mRNA transcripts by STAR[4], and only mapped reads were identified as exon transcripts. Besides, the MID sequences provided UMI information for transcript counting in which PCR duplications had been removed by handleBam[5] (https://github.com/BGIResearch/handleBam). Read pairs with the MID quality score lower than 10 were filtered out. Gene expression matrices with spatial information were eventually generated by Stereopy (https://github.com/BGIResearch/stereopy) using clean exonic data only.

Multimodal image registration

Registration of gene expression heatmaps to their corresponding ssDNA staining images was performed in a semi-automated manner. Considering the positional information in the anterior segment of the human eye is established at a cellular-subcellular resolution, we supposed that the registration should be possible at close to this resolution (< 10 µm) for real single-cell analyzes. Hence, we used an automated pipeline for the expression-ssDNA registration in a coarse-to-fine way. Specifically, as shown in Fig.S1B, we prepared and processed two image files as the input for registration. The carved track lines on the Stereo-seq chip surface provide precise marker information (~ 2-µm width) relevant for the registration optimization, where no RNAs are captured. Therefore, the greyscale image of ssDNA staining was first scaled according to the camera property, and the contrast was enhanced (https://github.com/BGI-Qingdao/GEM3D_toolkit). Gene expression matrices were converted to heatmap images, where each bin (500 nm) corresponds to a single pixel and the color magnitude represents the expression strength. Local contrast was again increased by integration of spatial expressions at different bin sizes. The whole-body and tissue morphology was used to roughly align two

datasets with TrakEM2 algorithm by Fiji[6]. This isotropically rigid registration including manual rotation, translation, scaling and mirror flipping was computed as a starting condition for the next fine registration. Notably, the morphological similarity was slightly disrupted due to RNA random diffusion during fixation and permeabilization processes, which might decrease the reliability of registration. Hence, the registration was further improved based on the track lines. An automated algorithm was used to associate track-line pairs and literately search the global maxima of pixel overlapping from paired track lines (https://github.com/BGI-Qingdao/GEM3D_toolkit). After the end of registration, a binary mask file from the aligned ssDNA-staining image was applied to remove transcripts outside the anatomical boundaries due to over-permeabilization. Finally, the ssDNA staining images and gene expression matrices (GEM profile) after registration were obtained.

Single-cell segmentation and assignment

Single cells can be segmented based on the locations of spliced and unspliced RNAs for the subcellular-resolution gene expression image by Stereo-seq[7]. Given that the segmentation is labor-intensive and complex, we considered to start from the segmentation of the nuclei by ssDNA staining image, and transferred the cell boundaries to the aligned gene expression image later in order to achieve the single-cell inferred level segmentation. Background noise of original ssDNA images was first filtered by a global threshold. Local contrast was adjusted with the CLAHE algorithm. A custom-programmed CellProfiler pipeline[8] was used to detect nuclei without manual annotation. Then the Mutex Watershed algorithm was used to automatically compute pixel affinities to predict for each pixel whether it belongs to this specific nucleus. At last, we assigned pinpoint RNA capturing spots to individual cells based on whether they fell within the segmented cell boundaries.

Cell dimensionality reduction, clustering and annotation

Based on the precise 2D gene expression image registration to the pairwise ssDNA-staining image, gene expression matrices were transformed into cell expression matrices following cell segmentation. For quality control purposes, we firstly excluded the cells which maintained the percentage of UMIs originating from mitochondrial and ribosomal genes below 20% for all chips. Additional statistics information on sequencing and analysis could be found in Table S2. Spatial gene expression profile rooted from different samples were conducted downstream analysis, respectively. Further analysis, including normalization, highly variable gene identification, dimension reduction, clustering, and differential expression analysis, were all performed using default parameters of SPATA2[9] (https://github.com/theMILOlab/SPATA2) except that dims were set at 1:30. Initially, 28 cell clusters from chip1 were obtained. And 29 cell clusters from chip 2 were obtained. To ensure reliable identification, cell clusters owning similar gene expression pattern per sample were combined. Finally, based on the canonical marker genes within AHCS reported by previous research[10-23], 15 cell clusters across different samples were identified (Table S3). In terms of subpopulation clustering, Integrative analysis of the TM cells from the two chips was performed using the Harmony function in Seurat[24]. Normalization, identification of highly variable gene, dimension reduction, clustering, and differential expression analysis, were all conducted using default parameters of Seurat V4[25]. Moreover, the H&E images were examined by professional pathologists to determine the tissue

types. The annotated Stereo-seq areas were confirmed to be consistent with H&E assessments and marker gene expression patterns.

IHC staining

Serial cryosections were prepared from the OCT-embedded tissue chips and stored at -80°C prior to use for IHC staining. Chips were warmed to room temperature, rinsed three times using PBS (pH 7.2, 5 min/wash). Chips were then processed with a kit (KIT-9710; MXB) based on provided directions. Briefly, they were treated for 40 min with Reagent 1 at 37°C to block endogenous peroxidase activity, washed three times with PBS as above, blocked for 30 min with Reagent 2 at 37°C, and probed overnight at 4°C with monoclonal anti-CFD (1:100; sc-47683; Santa Cruz Biotechnology) or anti-MYOC (1:500; 60357-1-Ig; Proteintech). Chips were then probed for 30 min with biotinylated secondary anti-mouse IgG (Reagent 3) for 30 min at 37°C, washed three times with PBS as above, incubated for 30 min with Reagent 4 at 37°C, and washed with PBS as above. DAB (DAB-0031; MXB) was then used for color development, and cells were treated with hematoxylin (CTS-1099; MXB) for 5 min at room temperature for nuclear counterstaining.

Differential gene expression and gene ontology (GO) enrichment analysis

Genes that were significantly up- or down-regulated were selected from subpopulation marker genes based on the following criteria: (1) |log2 fold-change| > 0.25 in expression levels; (2) genes were expressed in > 20% of cells associated with the target cluster; (3) adjusted $P < 0.05$. Subsequently, these genes were subjected to GO enrichment analyses performed with ClusterProfiler under the default[26]. Similar GO terms were merged and visualized using the treeplot() function according to the results of hierarchical clustering analyses.

Statistical analysis and visualization

All other statistical testing, including Wilcoxon signed-rank, and Wilcoxon rank-sum testing was also performed in R v4.1.0. $P < 0.05$ was the threshold for significance. The development of visual website was powered by VT3D[27].

Supplementary figure legends (see Additional File 1.pdf)

Fig.S1. Preprocessing and the sequencing statistic information of spatial transcriptome chips

(A) Schematic diagram of spatial transcriptome analysis for aqueous humor circulatory system;

(B) Flow diagram illustrating the pipeline of multimodal image registration and cell segment for reconstruction of spatially-resolved single-cell transcriptome. In the Step01, manual registration of gene expression heatmaps (top left) to their corresponding ssDNA staining images (bottom left) was performed. This whole progress was based on the alignment of tissue contour and the trackline in the Step02 (two pictures on the right); In the Step03, the ssDNA staining images after registration were utilized to detect the cell boundaries to achieve the inferred single cell (bottom right) segment;

(C) Boxplot of the number of captured transcripts (UMI counts) and genes (gene counts) of 4 samples rooted from 2 chips. More detailed sequencing information is shown in the Table S2.

Fig.S2. Cell annotation and regrouping in aqueous humor circulatory system (AHCS)

(A) Heatmap showing the mean expression level of marker genes among the cell clusters of AHCS;

(B) Spatial expression of cell clusters marker genes in AHCS. The color represents the mean expression levels of the marker genes;

(C) The spatial location of low-quality stromal cells in two chips (top two pictures). The boxplot showing the comparison of the number of captured transcripts (UMI counts) and genes (gene counts) of indicated cell clusters (bottom one picture) in chip1 and chip2, respectively.

Fig.S3. Subcluster annotation and analysis of cell specific expression pattern of disease-related genes

(A) Uniform manifold approximation and projection (UMAP) plot showing the distribution of two trabecular meshwork (TM) cell subclusters from different chips;

(B) UMAP projection colored by log-normalized gene expression levels of five signature genes specific for each of the two TM cell subclusters. *CFD, TYRP1* and *PMEL* are the markers for the anterior area of TM subclusters. *MYOC* and *ANGPTL7* are the markers for near-juxtacanalicular TM subcluster;

(C) The violin plot showing the normalized expression levels of five specific signature genes for TM subclusters;

(D) Immunohistochemistry of MYOC and CFD in the TM area (scale bars = 100 μm);

(E) Proportion distribution of 15 major cell types showing in bar plots from different chips (left panel) and total cell number of each cell type (right panel);

(F) Heatmap showing the expression of genes associated with glaucoma in each cell type;

(G) Heatmap demonstrating the expression of genes associated with four extracellular matrix components (collagens, fibronectin, laminins, elastin and fibrillin microfibrils) in each cell type.

Supporting Tables (see Additional File 2.xlsx)

Table S1. Clinical characteristics and experimental details for collected samples.

Table S2. Spatial transcriptome data statistics.

Table S3. Marker genes for the annotation of cell types in aqueous humor circulatory system.

Table S4. The enrichment analysis annotation of trabecular meshwork subpopulations.

Table S5. The disease-related genes collected from mendelian genes, risk factors in genome-wide association study (GWAS) and the online mendelian inheritance in man (OMIM) dataset.

Reference

1. Braunger BM, Fuchshofer R, Tamm ER. The aqueous humor outflow pathways in glaucoma: A unifying concept of disease mechanisms and causative treatment. *European journal of pharmaceutics and biopharmaceutics : official journal of Arbeitsgemeinschaft fur Pharmazeutische Verfahrenstechnik eV.* 2015;95(Pt B):173-181.
2. Johnstone M, Martin E, Jamil A. Pulsatile flow into the aqueous veins: manifestations in normal and glaucomatous eyes. *Experimental eye research.* 2011;92(5):318-327.
3. Li HL, Shan SW, Stamer WD, et al. Mechanistic Effects of Baicalein on Aqueous Humor Drainage and Intraocular Pressure. *International journal of molecular sciences.* 2022;23(13).
4. Dobin A, Davis CA, Schlesinger F, et al. STAR: ultrafast universal RNA-seq aligner. *Bioinformatics.* 2013;29(1):15-21.
5. Chen A, Liao S, Cheng M, et al. Spatiotemporal transcriptomic atlas of mouse organogenesis using DNA nanoball-patterned arrays. *Cell.* 2022;185(10):1777-1792.
6. Cardona A. TrakEM2: an ImageJ-based program for morphological data mining and 3d modeling. 2006.
7. Wei X, Fu S, Li H, et al. Single-cell Stereo-seq reveals induced progenitor cells involved in axolotl brain regeneration. *Science.* 2022;377(6610):eabp9444.
8. Carpenter AE, Jones TR, Lamprecht MR, et al. CellProfiler: image analysis software for identifying and quantifying cell phenotypes. *Genome Biol.* 2006;7(10):R100.
9. Ravi VM, Will P, Kueckelhaus J, et al. Spatially resolved multi-omics deciphers bidirectional tumor-host interdependence in glioblastoma. *Cancer Cell.* 2022;40(6):639-655.
10. Collin J, Hasoon MSR, Zerti D, et al. Single-cell RNA sequencing reveals transcriptional changes of human choroidal and retinal pigment epithelium cells during fetal development, in healthy adult and intermediate age-related macular degeneration. *Hum Mol Genet.* 2023;32(10):1698-1710.
11. Donadon M, Santoro MM. The origin and mechanisms of smooth muscle cell development in vertebrates. *Development.* 2021;148(7).
12. Lee MY, Park C, Berent RM, et al. Smooth Muscle Cell Genome Browser: Enabling the Identification of Novel Serum Response Factor Target Genes. *PLoS One.* 2015;10(8):e0133751.
13. Liu Z, Jin YQ, Chen L, et al. Specific marker expression and cell state of Schwann cells during culture in vitro. *PLoS One.* 2015;10(4):e0123278.
14. Patel G, Fury W, Yang H, et al. Molecular taxonomy of human ocular outflow tissues defined by single-cell transcriptomics. *Proceedings of the National Academy of Sciences.* 2020;117(23):12856-12867.
15. Tamm ER. The trabecular meshwork outflow pathways. Functional morphology and surgical aspects. *Glaucoma London: Saunders Elsevier.* 2009:31-44.
16. Taniguchi F, Suzuki Y, Kurihara H, et al. Molecular cloning of the bovine MYOC and induction of its expression in trabecular meshwork cells. *Invest Ophthalmol Vis Sci.* 2000;41(8):2070-2075.
17. Trimm E, Red-Horse K. Vascular endothelial cell development and diversity. *Nat Rev Cardiol.* 2023;20(3):197-210.

18. van Zyl T, Yan W, McAdams A, et al. Cell atlas of aqueous humor outflow pathways in eyes of humans and four model species provides insight into glaucoma pathogenesis. *Proceedings of the National Academy of Sciences.* 2020;117(19):10339-10349.
19. van Zyl T, Yan W, McAdams AM, Monavarfeshani A, Hageman GS, Sanes JR. Cell atlas of the human ocular anterior segment: Tissue-specific and shared cell types. *Proceedings of the National Academy of Sciences.* 2022;119(29):e2200914119.
20. Wang J, Rattner A, Nathans J. A transcriptome atlas of the mouse iris at single-cell resolution defines cell types and the genomic response to pupil dilation. *Elife.* 2021;10.
21. Watt B, van Niel G, Raposo G, Marks MS. PMEL: a pigment cell-specific model for functional amyloid formation. *Pigment Cell Melanoma Res.* 2013;26(3):300-315.
22. Wu C, Boey D, Bril O, et al. Single-cell transcriptomics reveals the identity and regulators of human mast cell progenitors. *Blood Adv.* 2022;6(15):4439-4449.
23. Gautron A, Migault M, Bachelot L, Corre S, Galibert MD, Gilot D. Human TYRP1: Two functions for a single gene? *Pigment Cell Melanoma Res.* 2021;34(5):836-852.
24. Korsunsky I, Millard N, Fan J, et al. Fast, sensitive and accurate integration of single-cell data with Harmony. *Nature methods.* 2019;16(12):1289-1296.
25. Hao Y, Hao S, Andersen-Nissen E, et al. Integrated analysis of multimodal single-cell data. *Cell.* 2021;184(13):3573-3587.
26. Yu G, Wang LG, Han Y, He QY. clusterProfiler: an R package for comparing biological themes among gene clusters. *Omics : a journal of integrative biology.* 2012;16(5):284-287.
27. Guo L, Li Y, Qi Y, et al. VT3D: a visualization toolbox for 3D transcriptomic data. *J Genet Genomics.* 2023;50(9):713-719.
