# Supplementary material for: Profiling the cell diversity and tissue structure of aqueous humor circulatory system in human eyes using spatial single-cell RNA sequencing
Source: Genes Dis. 2024 Apr 12;12(1):101304. doi: 10.1016/j.gendis.2024.101304 (PMC11472072; doi:10.1016/j.gendis.2024.101304)

Fig. S1

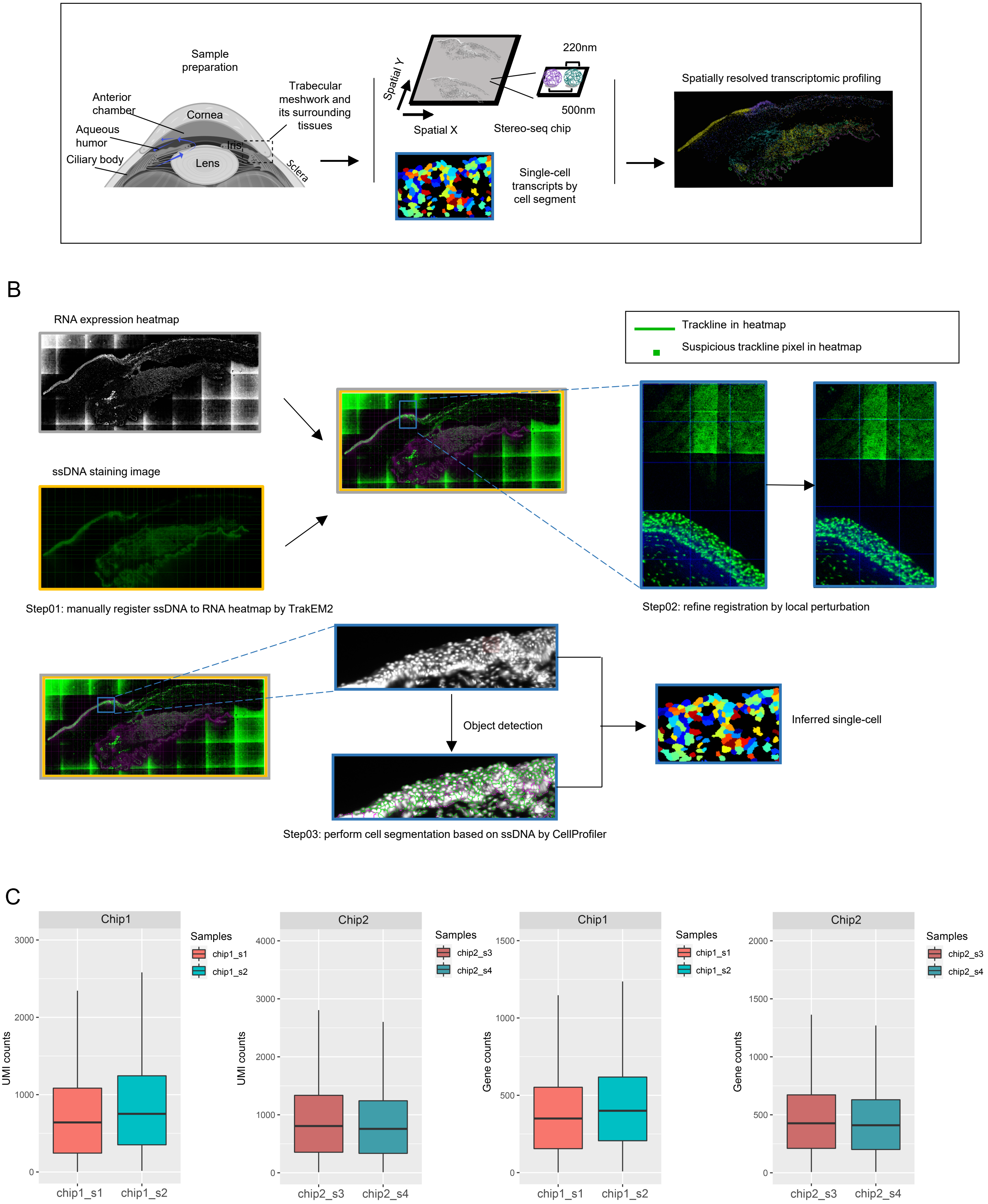
A
Sample preparation
Anterior chamber
Cornea
Aqueous humor
Iris
Ciliary body
Lens
Sclera
Trabecular meshwork and its surrounding tissues
Spatial Y
Spatial X
220nm
500nm
Stereo-seq chip
Single-cell transcripts by cell segment
Spatially resolved transcriptomic profiling
B
RNA expression heatmap
ssDNA staining image
Trackline in heatmap
Suspicious trackline pixel in heatmap
Step01: manually register ssDNA to RNA heatmap by TrakEM2
Step02: refine registration by local perturbation
Object detection
Inferred single-cell
Step03: perform cell segmentation based on ssDNA by CellProfiler
C
Chip1
Chip2
Samples
chip1_s1
chip1_s2
chip2_s3
chip2_s4
UMI counts
Gene counts
0
500
1000
1500
2000
3000
4000

Fig. S2

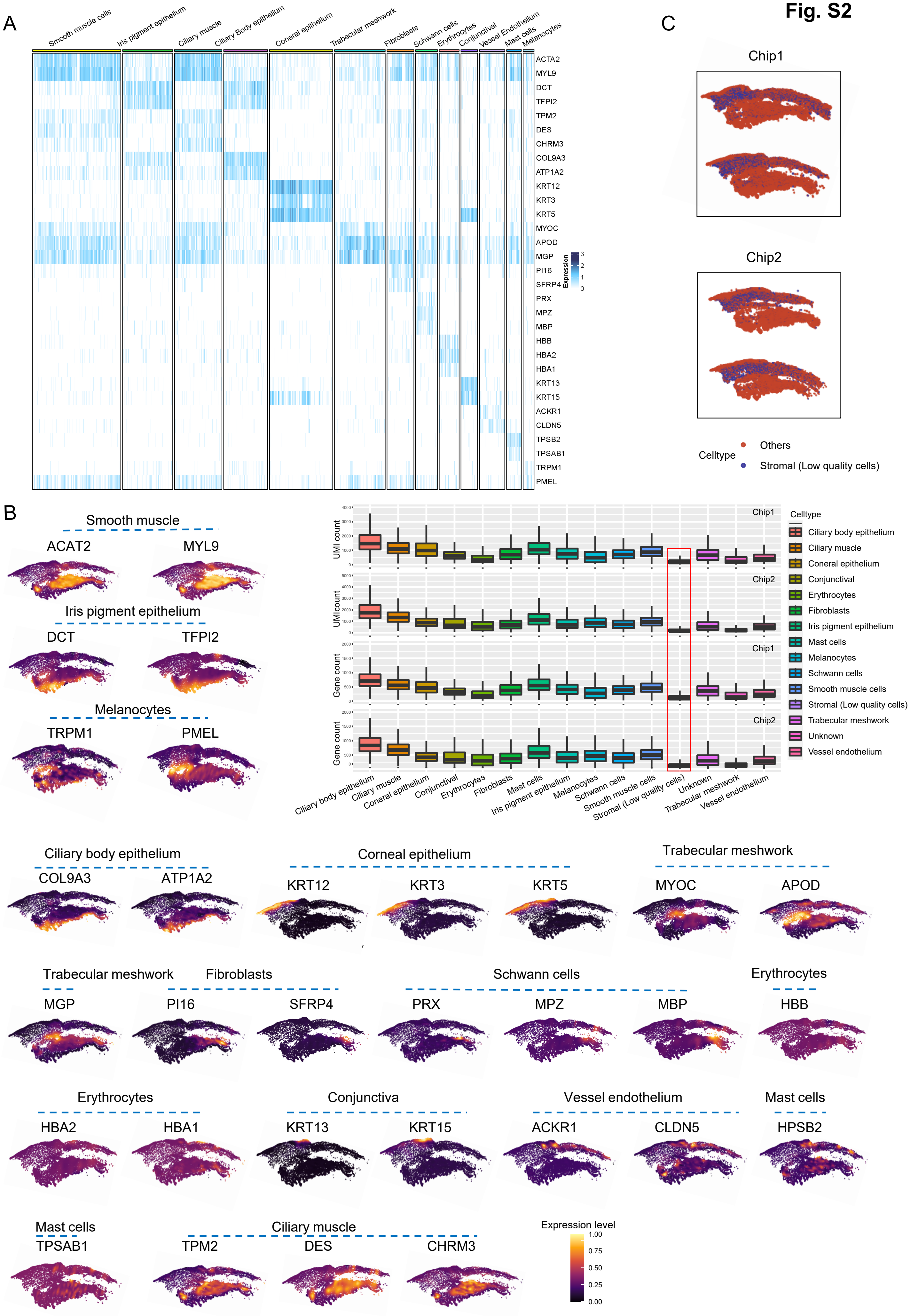
A
Smooth muscle cells
Iris pigment epithelium
Ciliary muscle
Ciliary Body epithelium
Coneral epithelium
Trabecular meshwork
Fibroblasts
Schwann cells
Erythrocytes
Conjunctival
Vessel Endothelium
Mast cells
Melanocytes
ACTA2
MYL9
DCT
TFPI2
TPM2
DES
CHRM3
COL9A3
ATP1A2
KRT12
KRT3
KRT5
MYOC
APOD
MGP
PI16
SFRP4
PRX
MPZ
MBP
HBB
HBA2
HBA1
KRT13
KRT15
ACKR1
CLDN5
TPSB2
TPSAB1
TRPM1
PMEL
Expression
3
2
1
0
C
Chip1
Chip2
Celltype
Others
Stromal (Low quality cells)
B
Smooth muscle
ACAT2
MYL9
Iris pigment epithelium
DCT
TFPI2
Melanocytes
TRPM1
PMEL
UMI count
UMIcount
Gene count
Chip1
Chip2
Chip1
Chip2
Celltype
Ciliary body epithelium
Ciliary muscle
Coneral epithelium
Conjunctival
Erythrocytes
Fibroblasts
Iris pigment epithelium
Mast cells
Melanocytes
Schwann cells
Smooth muscle cells
Stromal (Low quality cells)
Trabecular meshwork
Unknown
Vessel endothelium
Ciliary body epithelium
COL9A3
ATP1A2
Corneal epithelium
KRT12
KRT3
KRT5
Trabecular meshwork
MYOC
APOD
Trabecular meshwork
Fibroblasts
Schwann cells
Erythrocytes
MGP
PI16
SFRP4
PRX
MPZ
MBP
HBB
Erythrocytes
Conjunctiva
Vessel endothelium
Mast cells
HBA2
HBA1
KRT13
KRT15
ACKR1
CLDN5
HPSB2
Mast cells
Ciliary muscle
TPSAB1
TPM2
DES
CHRM3
Expression level
1.00
0.75
0.50
0.25
0.00

Fig. S3

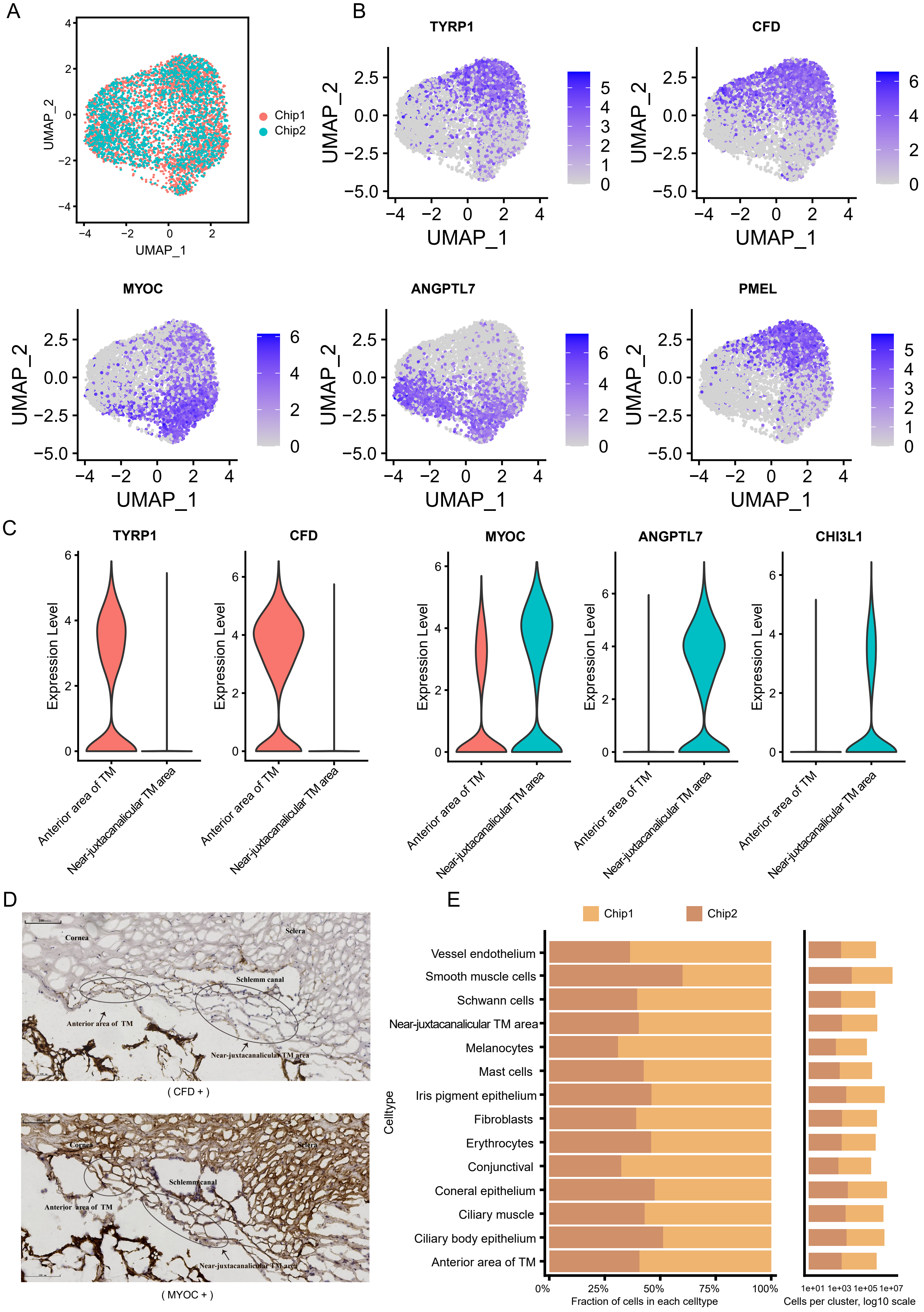

A
UMAP_1
UMAP_2
Chip1
Chip2
B
TYRP1
CFD
MYOC
ANGPTL7
PMEL
UMAP_1
UMAP_2
C
TYRP1
CFD
MYOC
ANGPTL7
CHI3L1
Expression Level
Anterior area of TM
Near-juxtacanalicular TM area
D
Cornea
Sclera
Schlemm canal
Anterior area of TM
Near-juxtacanalicular TM area
(CFD +)
Cornea
Sclera
Schlemm canal
Anterior area of TM
Near-juxtacanalicular TM area
(MYOC +)
E
Chip1
Chip2
Vessel endothelium
Smooth muscle cells
Schwann cells
Near-juxtacanalicular TM area
Melanocytes
Mast cells
Iris pigment epithelium
Fibroblasts
Erythrocytes
Conjunctival
Coneral epithelium
Ciliary muscle
Ciliary body epithelium
Anterior area of TM
Celltype
0%
25%
50%
75%
100%
Fraction of cells in each celltype
1e+01 1e+03 1e+05 1e+07
Cells per cluster, log10 scale

Fig. S3

F

Expression of Glaucoma-Associated Disease Gene

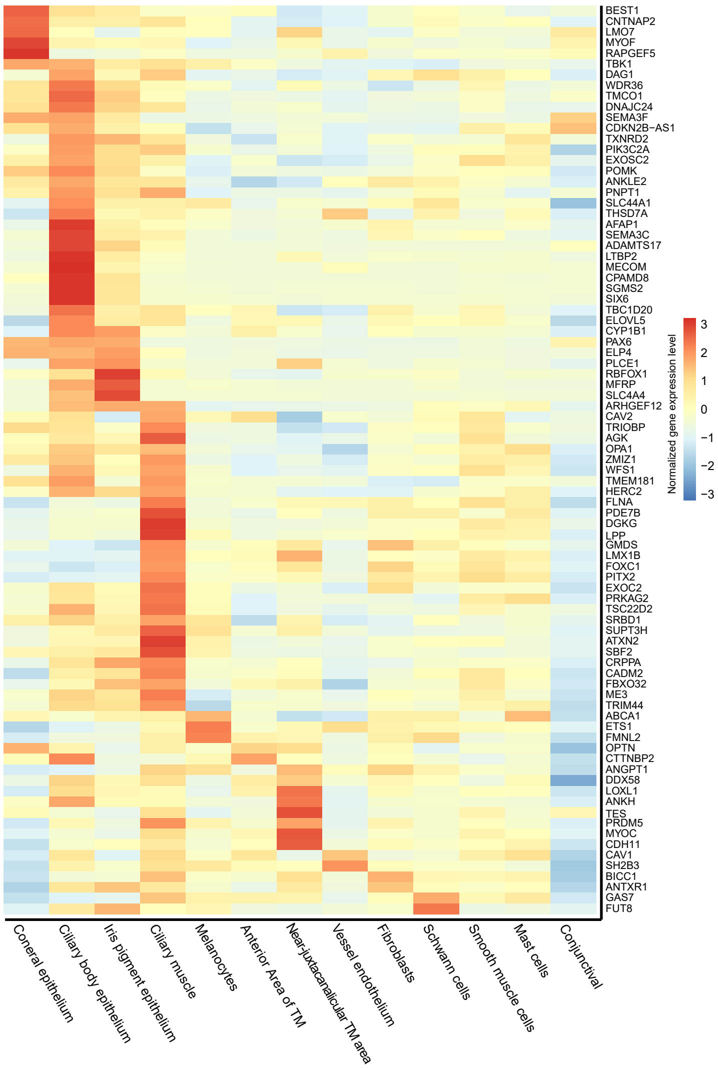

G

Expression of Collagens and Fibronectin -Associated Gene

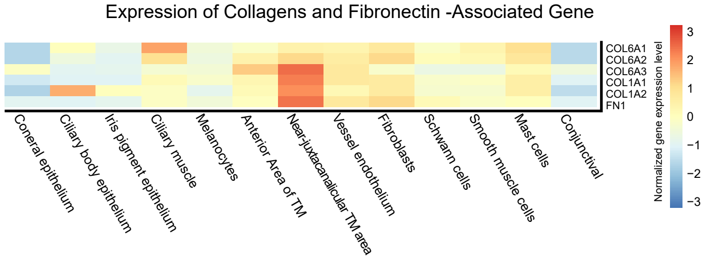

Expression of Laminins -Associated Gene

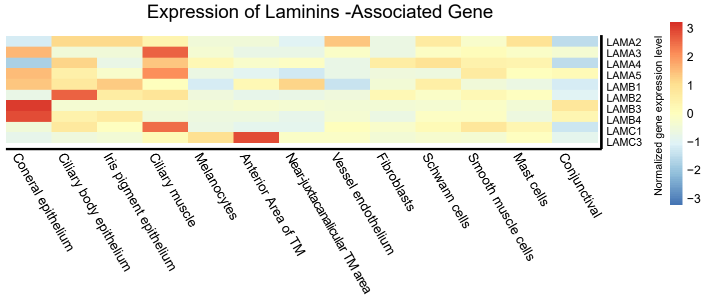

Expression of Elastin and Fibrillin Microfibrils -Associated Gene

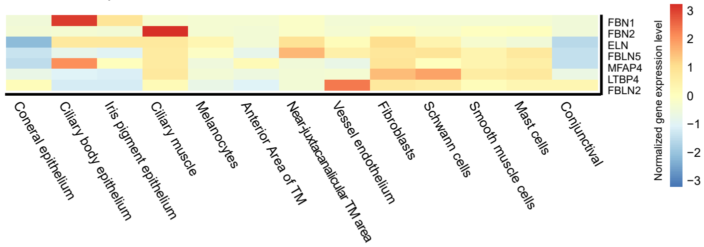

Supplement: Multimedia component 2 [file mmc2.pdf]
